# From Scar to Shunt: Incidental Discovery of Pulmonary Arteriovenous Malformation in a Healthy Adult Trauma Patient

**DOI:** 10.7759/cureus.92712

**Published:** 2025-09-19

**Authors:** Miriam Ifegwu, Savannah Aibaye, Genevieve Crawley, Fred Javdan

**Affiliations:** 1 College of Medicine, Ross University School of Medicine, Miramar, USA; 2 Department of Internal Medicine, California Hospital Medical Center, Los Angeles, USA

**Keywords:** asymptomatic hypoxemia, embolization therapy, hht, pavm, pulmonary arteriovenous malformation, trauma evaluation

## Abstract

Pulmonary arteriovenous malformations (PAVMs) are rare vascular anomalies that create right-to-left shunts, predisposing patients to hypoxemia, paradoxical emboli, and cerebral abscess. While often congenital and associated with hereditary hemorrhagic telangiectasia (HHT), they may also be acquired. We describe the case of a 59-year-old Hispanic man who was incidentally found to have a large left lower lobe PAVM with a 1.1 cm feeding artery during evaluation for traumatic injuries. Despite severe hypoxemic respiratory failure, he had no respiratory symptoms or distress. Initial embolization reduced but did not eliminate the shunt, necessitating a second procedure that achieved complete closure. This case reinforces the importance of recognizing that PAVMs can remain clinically silent despite severe physiologic burden and often require staged management when large feeding arteries are involved. Vigilant follow-up and genetic evaluation remain essential to preventing long-term complications.

## Introduction

Pulmonary arteriovenous malformations (PAVMs) are rare vascular connections between the pulmonary arteries and branches of the pulmonary veins that bypass the normal capillary network, resulting in a right-to-left shunt. This allows poorly oxygenated blood to enter the systemic circulation and predisposes patients to hypoxemia and paradoxical embolization, with potentially devastating neurologic sequelae if untreated. The estimated prevalence is approximately 1 in 2,600 individuals (95% confidence interval (CI) 1,315-5,555) [1-3]. Most PAVMs are congenital, with over 70% linked to hereditary hemorrhagic telangiectasia (HHT), a genetic disorder of vascular dysplasia, which involves mutations in angiogenesis-related genes such as Endoglin (ENG) and ACVRL1 (ALK1) [4]. Approximately 20% of PAVMs, however, are acquired in the setting of hepatic cirrhosis, schistosomiasis, Fanconi syndrome, infections, chronic pulmonary hypertension, hepatorenal syndrome, chest surgery, pregnancy, or penetrating chest trauma [4,5]. Anatomically, the majority are simple (single, direct connection), while complex (multiple arteries connecting to more than one segment) and multiple malformations (widespread involvement of lung segments) are less common [1,4-7].

Clinical manifestations vary from silent disease to exertional dyspnea, cyanosis, or hemoptysis. The likelihood and severity of symptoms are dependent on the lesion size, feeding artery diameter, and degree of hypoxemia. More specifically, it occurs when the PAVM exceeds 2 centimeters (cm), the feeding artery measures >3 millimeters (mm), or arterial oxygen pressure falls below 60 millimeters of mercury (mmHg) [2,4,7,8]. Serious complications if untreated are transient ischemic attack (TIA), stroke, and cerebral abscess from paradoxical embolization. Diagnosis of a PAVM requires a multimodal approach. Contrast-enhanced computed tomography (CT) and echocardiography with bubble study are the first-line tests, in which the latter grades shunt severity by microbubble count: Grade 1 (1-29), Grade 2 (30-100), and Grade 3 (>100), with higher grades correlating with neurologic risk [5,9]. Lung nuclear medicine perfusion scan (NM scan) serves as an adjunct test used to quantify shunt fraction, with >5% considered significant [10]. The right-to-left shunt ratio also serves as an index of response to treatment and prognostic value, and a large shunt significantly heightens morbidity and mortality risks [11,12]. CT angiography (CTA) is recommended prior to intervention to define the vascular anatomy, particularly when the feeding artery is 2-3 mm or more [10]. Despite this systematic approach, PAVMs may still be overlooked, especially in asymptomatic patients or in those without stigmata of HHT, where suspicion is low and incidental findings may be dismissed.

Treatment is recommended for asymptomatic patients with feeding arteries ≥2-3 mm, symptomatic non-pregnant patients, and symptomatic pregnant women in the second trimester [4,13]. Transcatheter embolization is the treatment of choice, with surgery reserved for refractory cases, those who are not candidates for embolization, or have failed prior treatment [14]. Recurrence or persistence of the malformation, which occurs in 25% of patients, necessitates long-term surveillance with follow-up CT scan at one month, one year, and every five years [15-17].

Here, we report the incidental discovery of a large, asymptomatic PAVM undergoing trauma evaluation. This case highlights the diagnostic challenge of PAVMs that remain clinically silent and the importance of maintaining vigilance when unexplained hypoxemia is encountered in unexpected settings. It also emphasizes the value of timely intervention because even asymptomatic patients without HHT can harbor clinically significant malformations.

## Case presentation

A 59-year-old Hispanic male with no significant medical history was brought to the emergency department by Emergency Medical Services (EMS) after falling from an electric scooter. The fall occurred as he swerved to avoid a car, landing on an outstretched hand and striking his face and head on the pavement.

The patient reported maintaining an active lifestyle, regularly running three to four miles without experiencing chest pain or shortness of breath. He denied any personal or family history of cardiovascular or pulmonary symptoms, PAVM, or HHT. He recalled an abnormal chest radiograph from his adolescence, although no further workup was pursued. Years later, during occupational tuberculosis screening, he again had an abnormal chest radiograph, described to him as a “scar,” which was deemed benign at the time.

**Figure 1:**
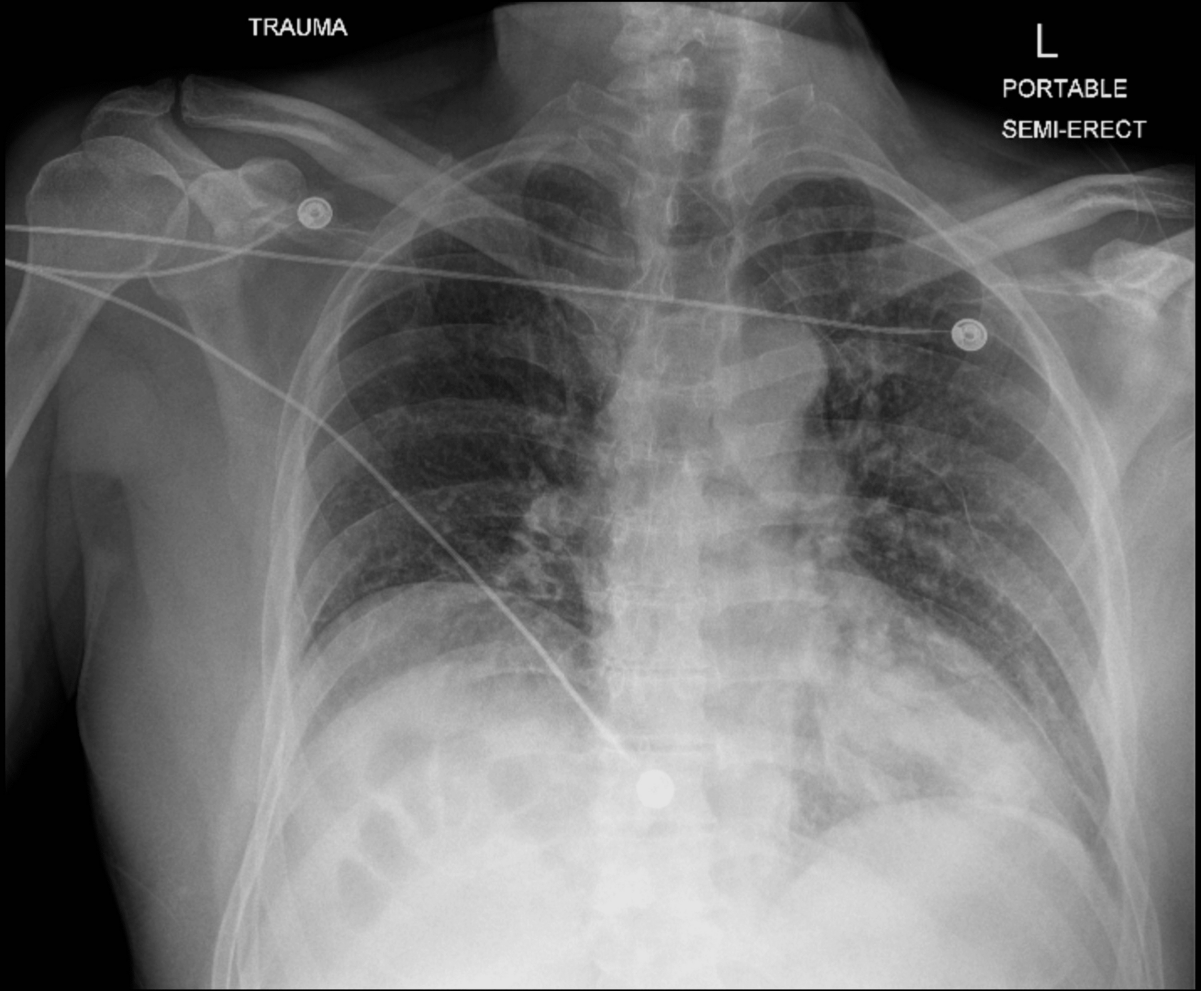
Frontal chest X-ray of a trauma patient showing no evidence of active cardiopulmonary disease.

On presentation, he endorsed only facial and right arm pain. He denied headache, chest pain, dyspnea, or neurological symptoms. Vital signs were stable except for an oxygen saturation of 87% on room air. Physical exam revealed facial swelling, hand tenderness, and chest wall discomfort without respiratory distress or adventitious breath sounds. Hemoglobin was 18.4 g/dL (normal 14-18 g/dL) and hematocrit 53.5% (normal 42-52%). Arterial blood gas (ABG) obtained due to persistent hypoxemia on room air revealed severe hypoxemic respiratory failure, with a PaO_2_/FiO_2_ (P/F) ratio of 80 (normal >300) and an Alveolar-arterial (A-a) gradient of 579 mmHg (expected normal for age: 18.5 mmHg), consistent with a significant right-to-left shunt. Chest X-ray was unremarkable (Figure 1), while other X-rays and maxillofacial CT revealed a mildly displaced intra-arterial fracture of the distal radius and communicated fracture of the right maxillary sinus with nasal bone fracture, respectively.

**Figure 2:**
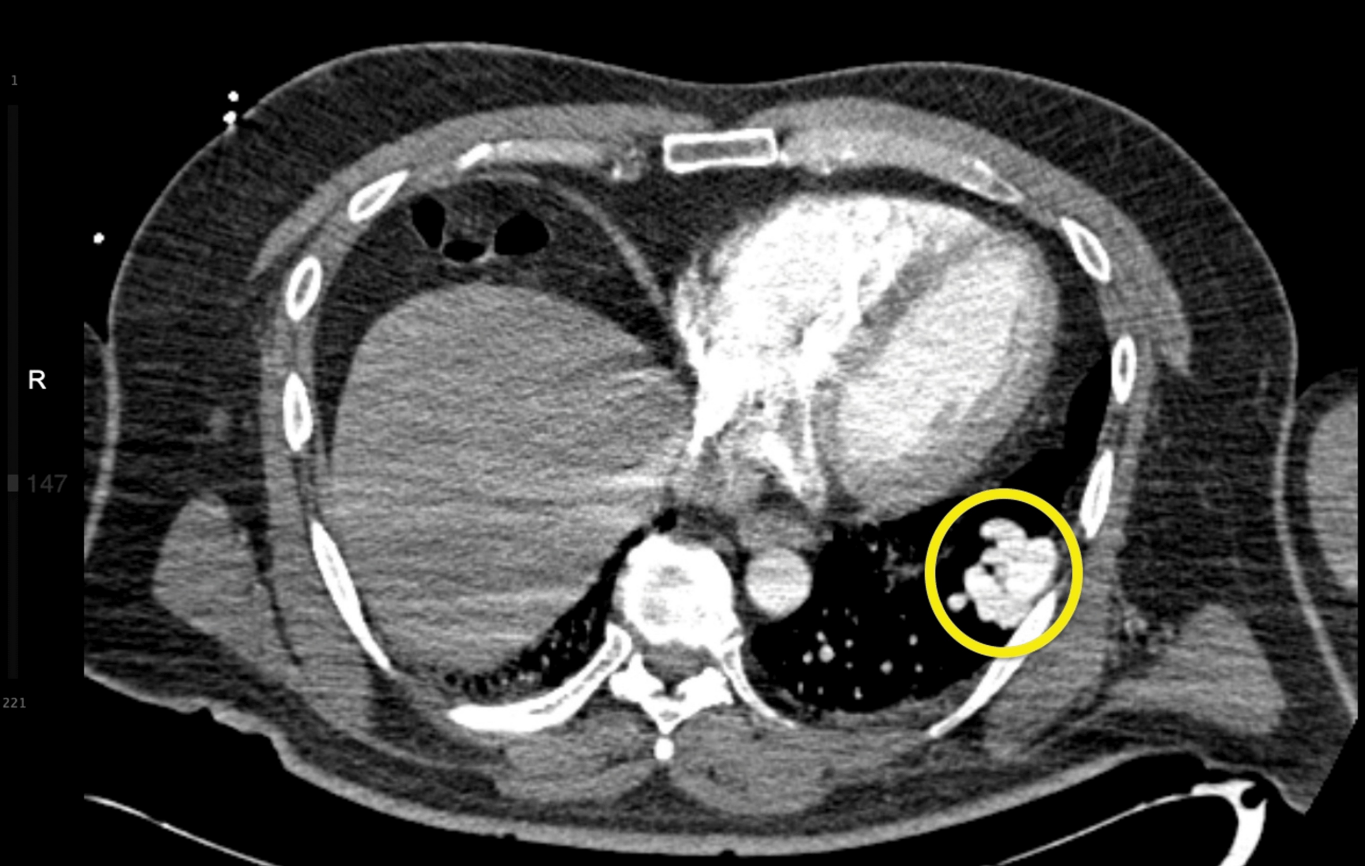
CT angiogram of chest (axial view) showing PAVM in the left lower lobe of the lung (3.5 cm x 6.8 cm) and feeding artery (1.1 cm) (yellow circle). PAVM: pulmonary arteriovenous malformation

**Figure 3:**
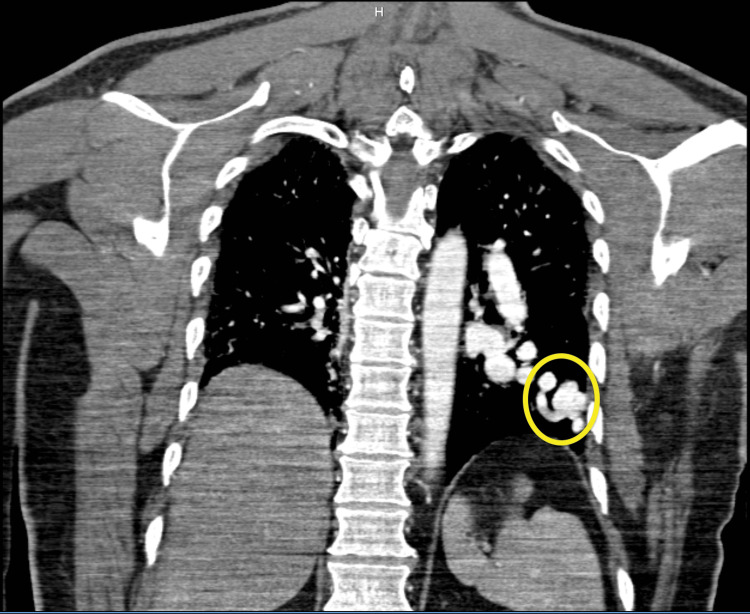
CT pulmonary angiogram (coronal view) showing a left lower lobe PAVM with perfusion defect (yellow circle). PAVM: pulmonary arteriovenous malformation

Given the severity of hypoxemia and suspicion for an intrapulmonary shunt, a contrast-enhanced chest CTA was obtained to evaluate for possible cardiac or pulmonary etiologies. The CTA revealed a 3.5 × 6.8 cm PAVM in the left lower lobe extending from the left infrahilar region to the pleura, with a 1.1 cm feeding artery (Figures 2-3).

**Figure 4:**
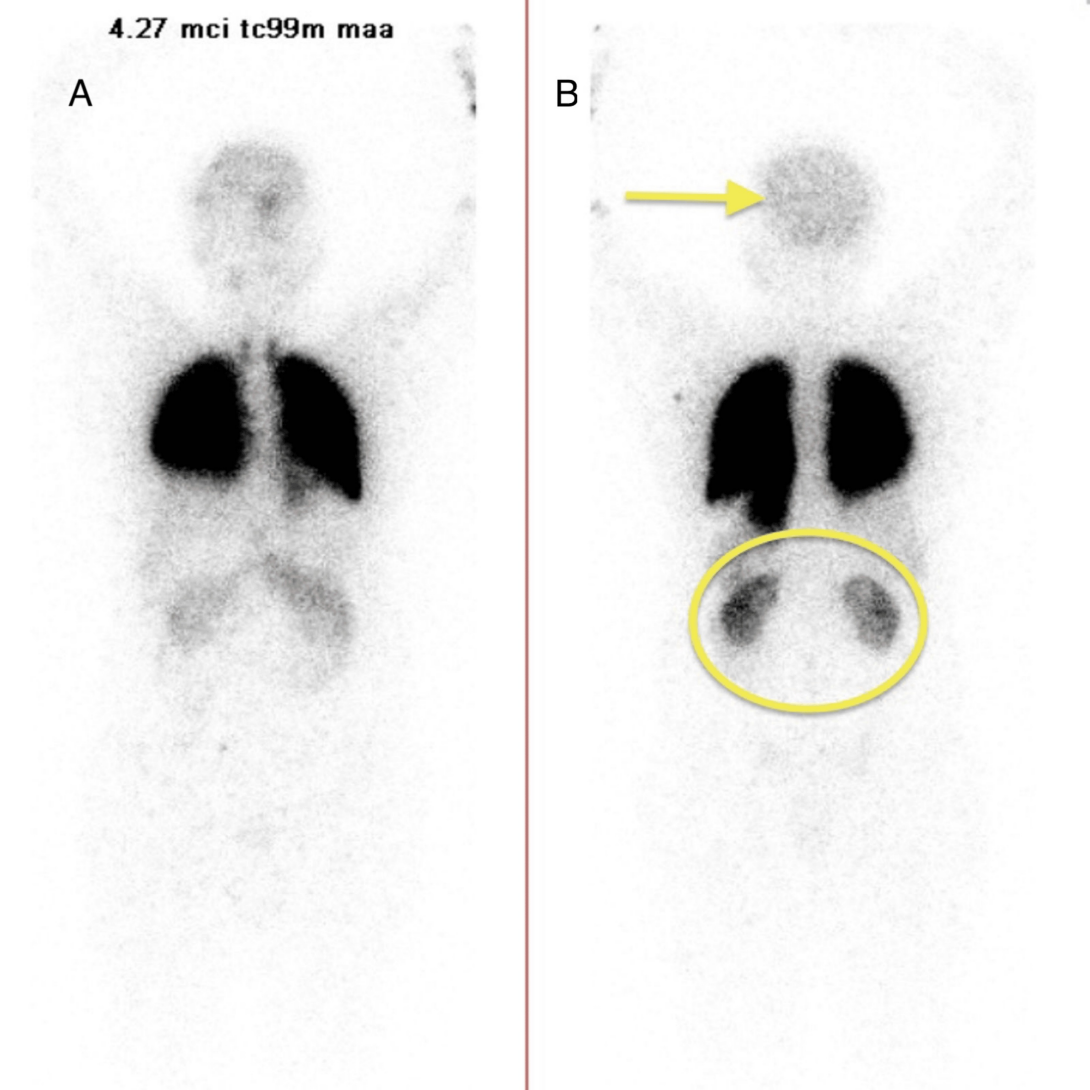
Pre-embolotherapy nuclear medicine (NM) lung perfusion scan showing expected uptake in lung bilaterally and increased uptake in brain, thyroid, spleen, kidney, and bowel, indicating a right-to-left (R-L) shunt. (A) Anterior view of NM lung perfusion scan; (B) posterior view of NM lung perfusion scan.

A bubble echocardiogram showed preserved ejection fraction (55-60%), trace pulmonic and tricuspid regurgitation, and a right-to-left shunt at the atrial septal level. Oxygen saturation improved to 92% with the use of supplemental oxygen via a nasal cannula. Afterwards, the patient was admitted for embolization. A pre-procedural NM scan demonstrated a right-to-left shunt ratio of 14% (Figure 4).

**Figure 5:**
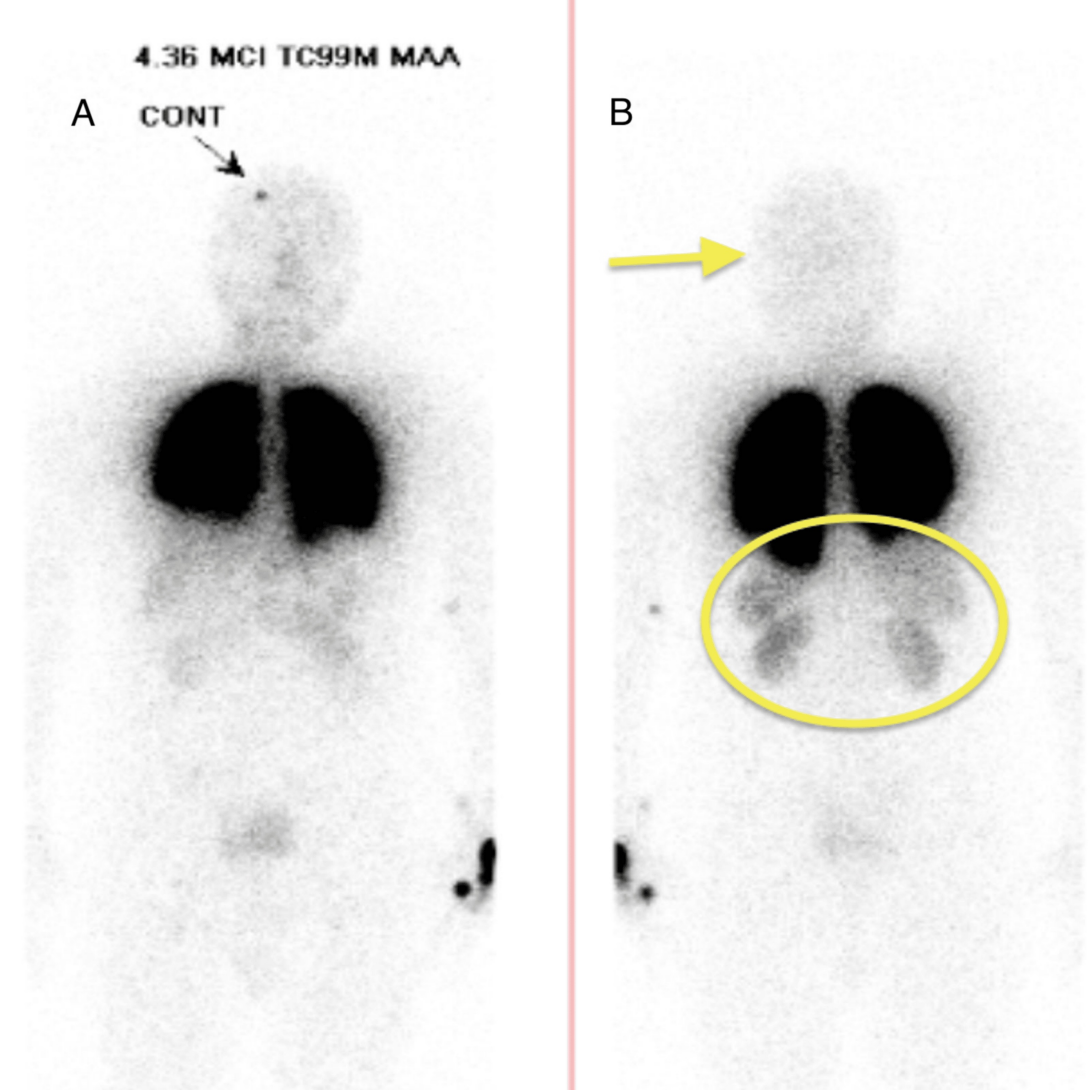
Post-embolization nuclear medicine (NM) lung perfusion scan showing improved brain perfusion but tracer uptake in spleen, kidney, and bowel, thus demonstrating residual shunt. (A) Anterior view of NM lung perfusion scan; (B) posterior view of NM lung perfusion scan.

On hospital day 3, the patient underwent embolization of the PAVM with nine detachable coils. However, the post-operative course was complicated by persistent hypoxemia with room air oxygen saturations ranging from 85% to 87%. Repeat NM scan showed improved shunt fraction of 3% with abnormal tracer uptake in the spleen, kidneys, and bowel (Figure 5).

Pulmonary angiography confirmed persistent flow through the coil mass into a large draining pulmonary vein and revealed high-grade stenosis of the left common and external iliac veins with collateralization.

On hospital day 6, a second embolization was performed with seven additional coils, achieving complete occlusion as confirmed by post-procedural angiography. The patient’s oxygenation improved, with oxygen saturation stabilizing between 92% and 94% on room air. His recovery was otherwise uncomplicated, and he was discharged in stable condition with referrals for pulmonology (for HHT genetic testing), otolaryngology, and orthopedic follow-up.

## Discussion

This case illustrates the incidental discovery and complex management of a large PAVM in a previously undiagnosed, asymptomatic individual, highlighting the challenges in diagnosis. The unusual context of discovery, combined with the discrepancy between lesion size and absence of symptoms, underscores the often silent nature of PAVMs, which may remain undetected for years and be discovered only during imaging for unrelated complaints.

PAVMs are rare abnormal connections between the pulmonary artery and branches of the pulmonary veins that bypass the capillary system. Most patients with PAVMs remain asymptomatic until the lesion exceeds 2 cm or when the feeding artery is greater than 3 mm, at which point symptoms such as exertional dyspnea, chest pain, or clubbing typically develop [2,4,7,8]. These features usually emerge as shunt volume increases and oxygenation declines. However, this case represents an atypical presentation as the patient demonstrated severe hypoxemic respiratory failure on ABG (P/F ratio of 80 and A-a gradient of 579), yet had no respiratory distress on examination. Despite having a sizable PAVM (3.5 × 6.8 cm) with a 1.1 cm feeding artery and a significant right-to-left shunt ratio of 14%, the patient remained highly active and asymptomatic. His tolerance of oxygen saturations in the mid-80s and lack of exertional limitations suggest long-standing physiologic adaptation, likely supported by compensatory erythrocytosis [7]. Notably, his history of abnormal chest radiographs in adolescence and later occupational screening, which were dismissed as benign, suggests that this lesion had likely been present for decades. Such misinterpretation as “scarring,” as occurred in this case, is a common diagnostic pitfall that can delay recognition and treatment of PAVMs. Although the patient reported no family history of PAVM or HHT, the absence of family history does not exclude the possibility of an inherited etiology, given the variable penetrance of HHT. Given the size of the lesion and its congenital appearance, outpatient genetic evaluation and screening for HHT were therefore recommended.

The initial embolization achieved partial improvement in oxygenation and a reduction in shunt fraction, but persistent flow remained. Residual shunting in PAVMs occurs in up to 25% of cases and results from incomplete occlusion of a large-caliber feeding artery, recruitment of collateral channels, technical limitations of coil deployment, or coil migration, well-documented challenges in PAVM management [15,16]. In this case, the large feeding artery (1.1 cm) likely contributed to incomplete occlusion despite the deployment of nine coils. NM scanning and angiography confirmed the residual shunt, necessitating a second intervention to fully occlude the feeding vessels, after which oxygenation improved and shunt flow resolved.

Post-procedure surveillance is essential to ensure complete closure and prevent complications. Current guidelines recommend follow-up CT scan at one month, one year, and every five years thereafter to detect recurrent or growth of untreated PAVMs [17]. In summary, this case demonstrates the variable clinical expression of PAVMs, the potential for long-term asymptomatic adaptation, and the importance of maintaining a broad differential when encountering unexplained hypoxemia, even in trauma settings. The partial response to initial embolization further underscores the technical challenges of treating large-caliber feeding arteries, which may require repeat intervention despite apparently adequate coil deployment. A systematic diagnostic approach and longitudinal follow-up remain essential to prevent long-term complications and guide appropriate genetic evaluation.

## Conclusions

This case illustrates how a potentially life-threatening PAVM was clinically silent for decades, even in the setting of severe respiratory failure, and was discovered incidentally during trauma evaluation. The paradox between the lesion size and absence of symptoms, coupled with partial failure of initial embolization, depicts both the silent nature of PAVMs and the technical challenges of management. It also reinforces the importance of maintaining clinical vigilance when confronted with unexplained hypoxemia, even in asymptomatic active and healthy patients. Once diagnosed, PAVMs generally require staged interventions and a multidisciplinary approach to achieve durable outcomes. Additionally, long-term follow-up and consideration of genetic evaluation remain critical components of care, particularly when congenital origins are suspected. Overall, identifying and treating PAVMs before major complications develop continues to be a significant challenge in clinical practice.
